# Correlation of prechemotherapy urinary megalin ectodomain (A-megalin) levels with the development of cisplatin-induced nephrotoxicity: a prospective observational study

**DOI:** 10.1186/s12885-019-6398-2

**Published:** 2019-12-02

**Authors:** Satoshi Shoji, Michihiro Hosojima, Hideyuki Kabasawa, Rie Kondo, Satoru Miura, Satoshi Watanabe, Nobumasa Aoki, Ryohei Kaseda, Shoji Kuwahara, Naohito Tanabe, Yoshiaki Hirayama, Ichiei Narita, Toshiaki Kikuchi, Hiroshi Kagamu, Akihiko Saito

**Affiliations:** 1 Department of Respiratory Medicine and Infectious Diseases, Niigata University Graduate School of Medical and Dental Sciences, 1-757 Asahimachi-dori, Chuo-ku, Niigata, Niigata 951-8510 Japan; 2 Department of Clinical Nutrition Science, Kidney Research Center, Niigata University Graduate School of Medical and Dental Sciences, 1-757 Asahimachi-dori, Chuo-ku, Niigata, Niigata 951-8510 Japan; 3 Present address: Department of Internal Medicine, Niigata Cancer Center Hospital, 2-15-3 Kawagishi-cho Chuo-ku, Niigata, Niigata 951-8566 Japan; 4 Department of Clinical Nephrology and Rheumatology, Kidney Research Center, Niigata University Graduate School of Medical and Dental Sciences, 1-757 Asahimachi-dori, Chuo-ku, Niigata, Niigata 951-8510 Japan; 5 Department of Applied Molecular Medicine, Kidney Research Center, Niigata University Graduate School of Medical and Dental Sciences, 1-757 Asahimachi-dori, Chuo-ku, Niigata, Niigata 951-8510 Japan; 6 Present address: Laboratory of Clinical Nutrition, Department of Nutrition, School of Human Cultures, The University of Shiga Prefecture, Hikone, Shiga Japan; 7 Department of Health and Nutrition, Faculty of Human Life Studies, University of Niigata Prefecture, 471 Ebigase, Higashi-ku, Niigata, Niigata 950-8680 Japan; 8 Reagent Research and Development Department, Denka Seiken Co., Ltd., 1-2-3 Minamihoncho, Gosen, Niigata, 959-1695 Japan; 9 Present address: Division of Respiratory Medicine, Saitama Medical University International Medical Center, 1397-1 Yamane, Hidaka, Saitama, 350-1298 Japan

**Keywords:** Chemotherapy, Cisplatin, Nephrotoxicity, Urinary megalin

## Abstract

**Background:**

Cisplatin is a potent chemotherapeutic agent used to treat a variety of solid tumors. One of the major side effects of cisplatin is dose-limiting nephrotoxicity. We recently demonstrated that the renal uptake of cisplatin and resultant cisplatin-induced nephrotoxicity are mediated in part by megalin, an endocytic receptor in proximal tubule epithelial cells (PTECs). We also developed sandwich enzyme-linked immunosorbent assays to measure the megalin ectodomain (A-megalin) and full-length megalin (C-megalin) in urine using monoclonal antibodies against the amino- and carboxyl-termini of megalin, respectively. The present study examined the correlation of urinary megalin level with cisplatin-induced nephrotoxicity and its utility as a biomarker in patients with thoracic cancer.

**Methods:**

This prospective observational study involved 45 chemotherapy-naïve patients scheduled to receive chemotherapy with ≥60 mg/m^2^ cisplatin for histologically diagnosed small cell lung cancer, non-small cell lung cancer, or malignant pleural mesothelioma. Before and after the first course of chemotherapy, we measured urinary A- and C-megalin and other markers of PTEC injury, such as *N*-acetyl-β-D-glucosaminidase, α_1_-microglobulin, β_2_-microglobulin, neutrophil gelatinase-associated lipocalin, and liver-type fatty acid-binding protein, and compared the values with the change in the estimated glomerular filtration rate (eGFR) and clinical risk factors for renal impairment.

**Results:**

A negative correlation was found between baseline urinary A-megalin levels and change in eGFR (*r* = − 0.458, *P* = 0.002). According to Kaplan–Meier survival curves, eGFR decline was associated with the baseline urinary A-megalin quartile (*P* = 0.038). In addition, according to the hazard ratios (HRs) for eGFR decline > 10 mL/min/1.73 m^2^ calculated using a Cox proportional hazard model, the highest quartile had a significantly higher risk of eGFR decline compared with the lowest quartile (HR 7.243; 95% confidence interval 1.545–33.962). Other baseline urinary markers showed no correlation with eGFR decline.

**Conclusions:**

This is the first report demonstrating that prechemotherapy urinary A-megalin levels are correlated with the development of cisplatin-induced nephrotoxicity. This finding has clinical implications for the identification of patients at risk for cisplatin-induced nephrotoxicity and the development of possible prophylactic therapies.

## Background

Cisplatin (*cis*-dichlorodiammineplatinum [II]) is one of the most potent chemotherapeutic agents used in the treatment of various solid tumors, including bladder cancer, cervical cancer, malignant pleural mesothelioma (MPM), ovarian cancer, squamous cell carcinoma of the head and neck, germ cell cancer, small cell lung cancer (SCLC), and non-small cell lung cancer (NSCLC). Cisplatin chemotherapy in combination with radiotherapy or surgery improves survival in advanced lung cancer and cure rate in locoregional and early stage lung cancer [1, 2].

The dose-limiting toxicity of cisplatin is renal, rather than hematological, typically causing acute kidney injury or even chronic renal impairment [3]. Cisplatin is thus contraindicated for chemotherapy in patients with chronic kidney disease, despite its outstanding efficacy. The kidneys are particularly vulnerable to toxic insult by cisplatin because of its high levels of accumulation in renal tissue [4]. Proximal tubule epithelial cells (PTECs) have been recognized as the primary target of cisplatin-induced toxicity [4]. Candidates for facilitated transport systems associated with cisplatin-induced nephrotoxicity include organic cation transporter 2 and copper transporter 1 in PTECs [5, 6]. However, an organic cation transporter 2 inhibitor, cimetidine, has shown only a partial protective effect against cisplatin-induced nephrotoxicity [5]. Thus, it is important to elucidate the precise mechanism underlying cisplatin-induced nephrotoxicity and develop strategies for its prediction and prevention.

Megalin is a large (~ 600 kDa) glycoprotein member of the low-density lipoprotein receptor family [7] that is expressed at the apical membranes of PTECs [8]. Megalin plays a pivotal role in the tubular reabsorption of glomerular filtrates and mediates intracellular signal transduction [9]. A high-fat-diet–induced mouse model of diabetic kidney disease showed that megalin mediates the proximal tubular uptake of nephrotoxic substances, such as lipid-modified proteins, resulting in tubuloglomerular alterations [10]. Megalin also mediates the uptake of nephrotoxic drugs such as aminoglycosides [11, 12], polymyxin B [11], colistin [12, 13], and vancomycin [12]. We demonstrated that cisplatin is a ligand of megalin using quartz crystal microbalance analysis. Moreover, we showed that PTECs expressing megalin develop specific cisplatin-induced injury in a mosaic megalin knockout mouse model, but that PTECs lacking megalin do not [12]. Therefore, megalin likely plays a primary role in the development of cisplatin-induced nephrotoxicity.

We also reported that two forms of megalin are excreted in urine, namely, the ectodomain (A-megalin) and full-length (C-megalin) forms, and that the former is several 100-fold more abundant than the latter [14]. We developed sandwich enzyme-linked immunosorbent assays (ELISAs) to measure urinary A- and C-megalin by using monoclonal antibodies (mAbs) against the amino- and carboxyl-termini of megalin, respectively [14]. Urinary C-megalin levels are increased via exocytosis from residual functioning nephrons that are overloaded by megalin-mediated protein metabolism [15]. In contrast, urinary A-megalin excretion appears to be regulated by the intracellular recycling [16] and intramembrane proteolysis [17–19] of megalin.

In this study, we measured urinary megalin levels, focusing in particular on A-megalin, in patients with advanced thoracic malignancies who were treated with cisplatin (≥60 mg/m^2^), with the aim of investigating the pathological role of megalin in the development of cisplatin-induced nephrotoxicity and elucidating the potential of urinary megalin measurement as a biomarker to predict nephrotoxicity.

## Methods

### Patients

Forty-five consecutive chemotherapy-naïve patients with Eastern Cooperative Oncology Group performance status of 0–1 and intact renal function (estimated glomerular filtration rate [eGFR] > 60 mL/min/1.73 m^2^) who were scheduled to receive chemotherapy with ≥60 mg/m^2^ cisplatin for histologically diagnosed NSCLC, SCLC, or MPM were enrolled at a single institution (Niigata University Medical and Dental Hospital, Niigata, Japan). This study was conducted in accordance with the Declaration of Helsinki and Good Clinical Practice guidelines and was approved by the Niigata University Ethics Committee. The patients were enrolled after providing written informed consent.

### Study design

On the day of the first course of chemotherapy (day 0), we obtained baseline serum creatinine (Cr) concentrations and urinary levels of A-megalin, C-megalin, *N*-acetyl-β-D-glucosaminidase (NAG), α_1_-microglobulin (α_1_-MG), β_2_-microglobulin (β_2_-MG), neutrophil gelatinase-associated lipocalin (NGAL), liver-type fatty acid-binding protein (L-FABP), and Cr. All patients received an intravenous infusion of ≥1500 mL isotonic electrolyte solution, supplemented with magnesium sulfate and diuretics (furosemide or mannitol). After antiemetic therapy consisting of 0.75 mg palonosetron, 9.9 mg dexamethasone, and the oral administration of 125 mg aprepitant, the patients received a 1-h intravenous infusion of cisplatin, followed by 1000 mL isotonic electrolyte solution. Fresh urine samples (20 mL) were scheduled to be collected on day 0 in the morning before cisplatin administration, and on days 1, 2, 4, 6, 7, 12, and 20 after administration. Urine samples were generally collected on the scheduled day ±1 or 2 days. Further urine sampling and measurement of biomarkers were performed at the discretion of the attending physicians. Urine samples were frozen at − 80 °C and thawed immediately prior to analysis. Serum Cr concentrations were routinely monitored after cisplatin administration (day 0 in the morning before cisplatin administration, and on days 2, 4, 7, 9, and 12 after administration). Serum Cr was generally collected on the scheduled day ±1 or 2 days. An adverse renal event was defined as eGFR decline > 10 mL/min/1.73 m^2^. This definition was based on a report that eGFR levels decrease by approximately 10 mL/min/1.73 m^2^ on average for each cycle of cisplatin treatment in cancer patients [20]. Smoking status and baseline therapies, using either renin-angiotensin system (RAS) inhibitors or non-steroidal anti-inflammatory drugs (NSAIDs), were confirmed from medical records. The presence of hypertension and diabetes was determined by clinical diagnosis.

### Measurement of human megalin in urine

Urinary A- and C-megalin were measured by ELISAs in the laboratory of Denka Co., Ltd., as described previously [14]. In brief, the capture mAbs (5 mg/mL) were immobilized on ELISA plates (LumiNunc F16 Maxisorp Surface Plate; Thermo Fisher Scientific, Inc., Waltham, MA) at 4 °C overnight. The Fab′ fragments of the tracer mAbs were conjugated to alkaline phosphatase (Roche Diagnostics, GmbH, Mannheim, Germany). Urine samples (90 μL) were mixed with 10 μL solution A (2 mol/L Tris-HCl, 0.2 mol/L ethylenediaminetetraacetic acid, 10% Triton X-100; pH 8.0) and incubated for 1 min at room temperature for the C-megalin assay, or for 3 h at 50 °C for the A-megalin assay, and reacted with alkaline phosphatase-labeled tracer mAbs in the ELISA plates. Urinary megalin concentrations were standardized by adjustment to urinary Cr concentrations.

### Measurement of other markers

Plasma concentrations of Cr were measured by an enzymatic method in the clinical laboratory at Niigata University Medical and Dental Hospital. Urinary concentrations of Cr, NAG, α_1_-MG, and β_2_-MG were measured with an automated instrument (7170S; Hitachi High-Technologies Corp., Tokyo, Japan) using the reagent kits CRE-S (Denka Seiken Co., Ltd., Gosen, Japan), N-assay L NAG NITTOBO (Nittobo Medical Co., Ltd., Tokyo, Japan), aMi-Latex (Denka Seiken Co., Ltd.), and BMG-Latex (Denka Seiken Co., Ltd.), respectively, at the laboratory of Denka Co., Ltd. Urinary NGAL was measured on the Abbott ARCHITECT i1000sr system at the laboratory of SRL Co., Ltd. (Tokyo, Japan). L-FABP was measured with a High Sensitivity Human L-FABP ELISA Kit (CMIC Holdings Co., Ltd., Tokyo, Japan) in our laboratory. The concentrations of each urinary marker were normalized to those of urinary Cr (/g Cr). eGFR was calculated using an equation that has been validated for the Japanese population [21]. ΔeGFR and Δurinary markers were calculated as follows: ΔeGFR (mL/min/1.73 m^2^) = (minimum eGFR after cisplatin administration) – (eGFR before cisplatin administration); Δurinary marker = (maximum urinary marker after cisplatin administration) – (urinary marker before cisplatin administration).

### Statistical analysis

The subjects were classified into 4 groups according to quartile of baseline urinary A-megalin. Numerical data are expressed as the mean ± standard deviation, and categorical data are expressed as *n* (%). Differences between groups were tested by one-way analysis of variance for numerical variables and the chi-square test for categorical variables. The correlation between two numerical variables was examined using Pearson’s correlation coefficient (*r*). Event-free survival curves were drawn using the Kaplan–Meier method, and differences between curves were tested by log-rank test. Mean differences in ΔeGFR according to quartile of urinary A-megalin were estimated using crude and adjusted models of linear regression analysis with the lowest quartile (Q1) as the reference. The adjusted model was adjusted for baseline eGFR. For these models, *P*-values for trend were also calculated using the quartile median values. Cox proportional hazards regression analysis was used to calculate crude and adjusted hazard ratios (HRs) and 95% confidence intervals (CIs) for the first adverse renal event according to baseline urinary A-megalin quartiles. All statistical analysis was performed using SPSS v.18.0 (IBM Corp., Armonk, NY). The level of significance was two-tailed *P* < 0.05.

## Results

The baseline characteristics of the subjects according to quartile of baseline urinary A-megalin are shown in Table 1. There were no significant differences between the groups in age, sex, smoking history, use of RAS inhibitors or NSAIDs, presence of hypertension or diabetes mellitus, baseline eGFR, and cisplatin dose. Tumor types were NSCLC (*n* = 33), SCLC (*n* = 10), and MPM (*n* = 2). Concomitant chemotherapeutic agents included pemetrexed (*n* = 18), etoposide (*n* = 12), gemcitabine (*n* = 9), CPT-11 (*n* = 3), docetaxel (*n* = 2), and vinorelbine (*n* = 1). Seven patients received the antivascular endothelial growth factor mAb bevacizumab with cisplatin and pemetrexed (Additional file 1: Table S1).

**Table 1:** Baseline characteristics of all study patients (*n* = 45)

	All	Q1	Q2	Q3	Q4	*P*
	*n* = 45	*n* = 11	*n* = 12	*n* = 11	*n* = 11	
Urinary A-megalin (pmol/g Cr)						
Mean ± SD or [Median]	87.9 ± 46.6	[36.1]	[76.0]	[95.8]	[150.4]	
Range	1.6–203.6	1.6–52.9	68.3–79.8	79.9–118.9	119.8–203.6	
Age, years						
Mean ± SD	64.6 ± 8.2	62.1 ± 8.9	65.8 ± 4.9	65.5 ± 8.4	64.8 ± 10.3	0.702
Sex, *n* (%)						0.399
Female	9 (20.0)	3 (27.3)	4 (33.3)	1 (9.1)	1 (9.1)	
Male	36 (80.0)	8 (72.7)	8 (66.7)	10 (90.9)	10 (90.9)	
Body height, cm						
Mean ± SD	163.9 ± 7.2	165.0 ± 6.9	161.3 ± 6.0	165.2 ± 7.0	164.5 ± 8.9	0.528
Body weight, kg						
Mean ± SD	57.7 ± 10.1	60.2 ± 10.2	59.9 ± 12.8	54.3 ± 10.6	56.0 ± 4.9	0.437
Smoking status, *n* (%)						0.527
Current/former	40 (88.9)	10 (90.9)	10 (83.3)	11 (100)	9 (81.8)	
Never	5 (11.1)	1 (9.1)	2 (16.7)	0 (0.0)	2 (18.2)	
Baseline therapies, *n* (%)						
RAS inhibitors	10 (22.2)	3 (27.3)	2 (16.7)	4 (36.3)	1 (9.1)	0.457
NSAIDs	22 (48.9)	5 (45.5)	5 (45.5)	4 (36.3)	8 (72.7)	0.340
Baseline comorbidity, *n* (%)						
Hypertension	20 (44.0)	3 (27.3)	9 (75.0)	5 (45.5)	3 (27.3)	0.066
Diabetes	9 (20.0)	1 (9.1)	2 (16.7)	3 (27.3)	3 (27.3)	0.675
Initial eGFR (mL/min/1.73 m^2^)						0.372
Mean ± SD	89.7 ± 15.9	83.7 ± 16.2	87.9 ± 17.1	92.9 ± 17.6	94.6 ± 11.9	
≧90	24 (53.3)	4 (36.4)	8 (66.7)	6 (54.5)	6 (54.5)	
60–89	20 (44.4)	7 (63.6)	3 (25.0)	5 (45.5)	5 (45.5)	
≦59	1 (2.2)	0 (0.0)	1 (8.3)	0 (0.0)	0 (0.0)	
Cisplatin dose (mg/m^2^)						
Mean ± SD	76.0 ± 5.0	73.2 ± 6.8	77.5 ± 2.6	75.9 ± 5.8	77.3 ± 2.6	0.143
Type of malignancy, *n* (%)						
NSCLC	33					
SCLC	10					
MPM	2					

eGFR, estimate glomerular filtration rate; Cr, creatinine; MPM, malignant pleural mesothelioma; NSAID, non-steroidal anti-inflammatory drug; NSCLC, non-small cell lung cancer; RAS, renin-angiotensin-aldosterone system; SCLC, small cell lung cancer; SD, standard deviation

We previously reported that urinary A- and C-megalin levels in healthy control individuals were 73 (35–106) and 0.145 (0.187–0.233) pmol/g Cr, respectively [14]. Mean baseline urinary A-megalin in the present study population (87.9 ± 46.6 pmol/g Cr) was nearly equal to that in our previous report, and mean baseline urinary C-megalin in the present study (0.64 ± 0.76 pmol/g Cr) was slightly higher (Additional file 1: Table S2). Mean NAG, α_1_-MG, β_2_-MG, NGAL, and L-FABP are also shown in Additional file 1: Table S2.

**Fig. 1:**
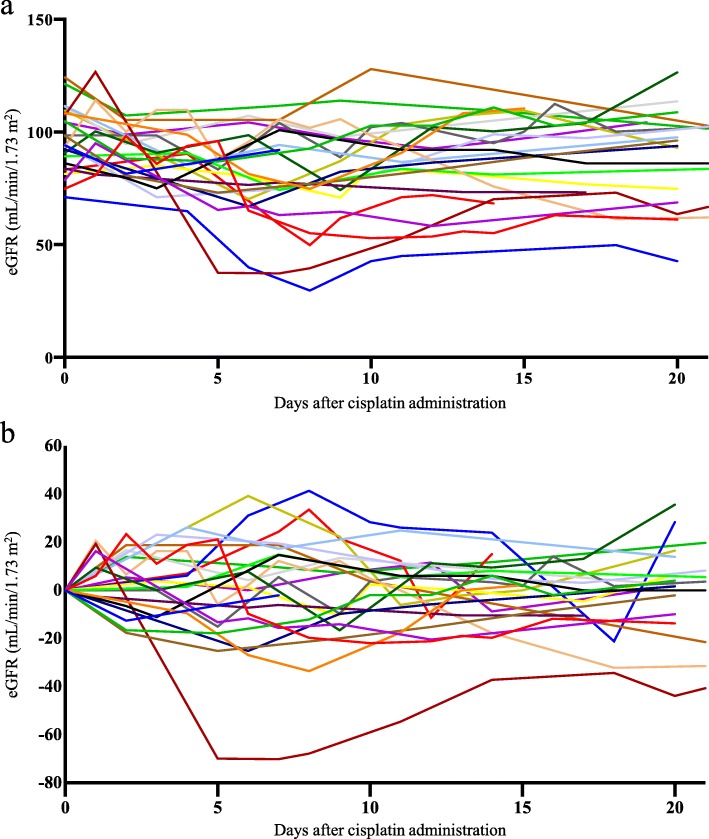
eGFR levels after cisplatin administration in 24 cases with an adverse renal event. The vertical axis represents the absolute value of eGFR (**a**) and the change from baseline eGFR (**b**), respectively

During 564 person-days of follow-up, 24 cases (53.3%) experienced adverse renal events (Fig. 1); the incidence rate of the first event was 0.426 per 10 person-days. Of those patients, the mean follow-up period until the first event was 5.8 ± 3.5 days from the start of cisplatin treatment. We found no association between pemetrexed administration and nephrotoxicity (data not shown).

**Fig. 2:**
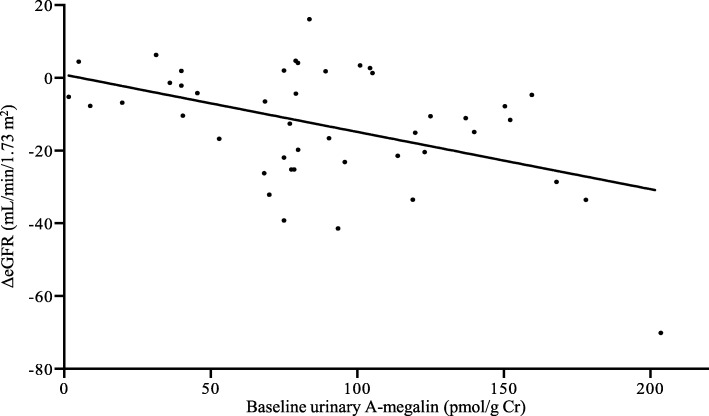
Scatter diagram of the relationship between ΔeGFR and baseline urinary A-megalin levels. ΔeGFR (mL/min/1.73 m^2^) = (minimum eGFR after cisplatin administration) – (eGFR before cisplatin administration). *r* = − 0.458, *P* = 0.002

**Fig. 3:**
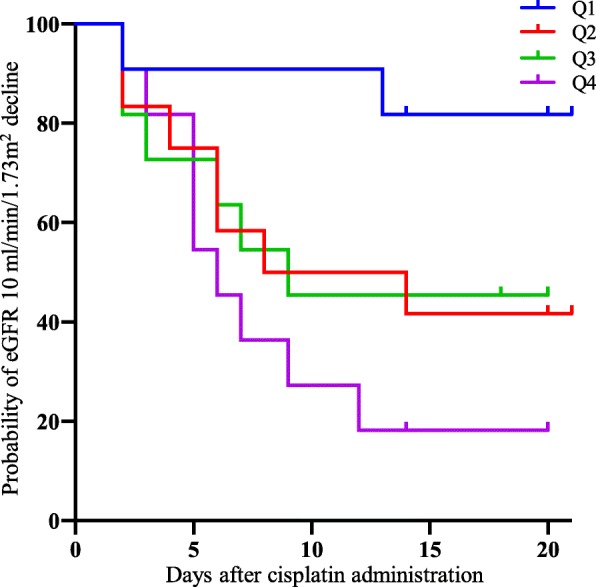
Kaplan–Meier curves for adverse-renal-event–free survival. An adverse renal event was defined as eGFR decline > 10 mL/min/1.73 m^2^. Tick marks indicate censored observations. Baseline higher urinary A-megalin levels tended to be associated with poorer adverse-renal-event–free survival (*P* = 0.038, log-rank test)

According to Pearson’s correlation coefficients, the baseline values of urinary A-megalin and eGFR showed negative correlations with ΔeGFR (*r* = − 0.458, *P* = 0.002 and *r* = − 0.395, *P* = 0.007, respectively): the correlation matrixes are shown in Table 2 and Additional file 1: Table S3, and the scatter diagram is shown in Fig. 2. In addition, A-megalin levels were not correlated with other urinary markers, including C-megalin, at baseline. The other urinary markers did not show any correlation with ΔeGFR. The adverse-renal-event–free Kaplan–Meier survival curves showed that higher baseline urinary A-megalin levels tended to be associated with poorer outcome (*P* = 0.038, log-rank test) (Fig. 3). By quartile of A-megalin, risk of eGFR decline was lowest in Q1, intermediate in Q2 and Q3, and highest in Q4.

**Table 2:** Correlations between renal function and urinary markers

	eGFR		ΔeGFR	
	*r*	*P*	*r*	*P*
eGFR	–		−0.395	0.007
ΔeGFR	−0.395	0.007	–	
A-megalin	0.299	0.046	−0.458	0.002
ΔA-megalin	0.241	0.111	−0.242	0.110
C-megalin	0.043	0.780	− 0.208	0.171
ΔC-megalin	0.075	0.623	−0.179	0.239
NAG	−0.260	0.864	−0.142	0.352
ΔNAG	−0.092	0.546	−0.235	0.120
α_1_-MG	−0.065	0.669	−0.287	0.056
Δα_1_-MG	−0.023	0.880	0.231	0.126
β_2_-MG	−0.142	0.352	−0.048	0.757
Δβ_2_-MG	−0.248	0.100	−0.038	0.802
NGAL	−0.071	0.642	0.124	0.417
L-FABP	0.123	0.421	−0.074	0.629

*r*, Pearson’s correlation coefficient. α_1_-MG, α_1_-microglobulin; β_2_-MG, β_2_-microglobulin; eGFR, estimated glomerular filtration rate; L-FABP, liver-type fatty acid-binding protein; NAG, *N*-acetyl-β-D-glucosaminidase; NGAL, neutrophil gelatinase-associated lipocalin. ΔeGFR, maximum change from the baseline value to the lowest value of eGFR during follow-up. Δurinary marker = (maximum urinary marker after cisplatin administration) – (urinary marker before cisplatin administration). NGAL and L-FABP were measured only before cisplatin administration

When mean ΔeGFR was compared between the quartiles of baseline urinary A-megalin, the decline in eGFR was greater in the highest quartile (Q4) than in the lowest quartile (Q1): the adjusted mean difference in ΔeGFR between Q4 and Q1 was − 13.27 mL/min/1.73 m^2^ (95% CI, − 26.168 to − 0.374, *P* = 0.012) (Table 3). The elevated risk of an adverse renal event associated with elevated A-megalin levels (Q4 vs. Q1) was significant in the crude model and marginally significant in the adjusted model (adjusted HR, 4.39; 95% CI, 0.911 to 21.134, *P* = 0.065).

**Table 3:** Difference in ΔeGFR and the risk of adverse renal events according to quartile of urinary A-megalin

	Quartile of urinary A-megalin								*P*
	Q1 (*n* = 11)		Q2 (*n* = 12)		Q3 (*n* = 11)		Q4 (*n* = 11)		
ΔeGFR (mL/min/1.73 m^2^)	−3.8	±6.7	−15.2	±15.1	−11.9	±18.0	−20.8	±18.5	0.085^a^
Estimated difference (95% CI) from Q1 (mL/min/1.73 m^2^)									
Crude	0.00	(reference)	−11.39	(−24.30, 1.51)^+^	−8.05	(−21.23, 5.14)	−16.95	(−30.13, −3.76)*	0.018^b^
Adjusted	0.00	(reference)	−10.00	(−22.29, 2.38)	−4.94	(−17.73, 7.85)	−13.27	(−26.17, −0.37)*	0.064^b^
Adverse renal events / person-days	2	/ 194	7	/ 146	6	/ 129	9	/ 95	
Event rate (/10 person days)	0.103		0.479		0.465		0.947		
Hazard ratio (95% CI)									
Crude	1.00	(reference)	4.25	(0.88, 20.51)^+^	4.04	(0.81, 20.09)^+^	7.24	(1.55, 33.96)*	0.008^b^
Adjusted	1.00	(reference)	3.80	(0.78, 18.48)^+^	3.03	(0.60, 15.30)	4.39	(0.91, 21.13)^+^	0.093^b^

CI, confidence interval; eGFR, estimated glomerular filtration rate. ΔeGFR, maximum change from the baseline value to the lowest value of eGFR during follow-up. An adverse renal event was defined as an eGFR decline of > 10 mL/min/1.73 m^2^. Adjusted hazard ratio, adjusted for baseline eGFR. ^+^*P* < 0.1, *P < 0.05, ^a^*P* for statistical difference between groups, ^b^*P* for trend

## Discussion

We examined the ectodomain (A-megalin) and full-length (C-megalin) forms of megalin in urine as markers of cisplatin-induced nephrotoxicity and found that prechemotherapy urinary A-megalin levels were associated with the development of cisplatin-induced nephrotoxicity. This is the first report to describe a relationship between prechemotherapy A-megalin levels and cisplatin-induced nephrotoxicity and to demonstrate that prechemotherapy A-megalin levels may be useful for predicting cisplatin-induced nephrotoxicity.

We previously reported that urinary C-megalin levels are correlated with severity of diabetic kidney disease [14] and IgA nephropathy [22]. The mechanism underlying urinary C-megalin excretion is associated with exocytosis, based on the chronic lysosomal protein metabolic load on PTECs of residual functioning nephrons [15]. However, baseline urinary C-megalin, NAG, α_1_-MG, β_2_-MG, NGAL, and L-FABP showed no correlation with the development of cisplatin-induced nephrotoxicity, suggesting that the mechanisms underlying urinary excretion of C-megalin and the other markers are not primarily associated with the pathogenesis of cisplatin-induced nephrotoxicity, at least in the patients enrolled in the present study.

In contrast, urinary A-megalin excretion was not correlated with other urinary markers including C-megalin and appears to be regulated by a distinct mechanism. Megalin undergoes intracellular recycling in PTECs and metalloprotease-mediated ectodomain shedding by regulated intramembrane proteolysis [17, 18]. Thus, urinary A-megalin may be produced as a normal byproduct in the absence of PTECs damage and may be increased by some factors that accelerate intracellular recycling. The efficiency of megalin recycling is also associated with its endocytic function [16]. Hence, it is likely that baseline urinary A-megalin is correlated with the amount and/or endocytic rate of megalin expressed in PTECs. It remains to be determined what factors are involved in the regulation of megalin recycling and/or endocytosis. We recently reported that megalin-mediated cisplatin uptake in PTECs has a primary role in nephrotoxicity [12]. Thus, baseline urinary A-megalin might reflect the efficiency of megalin-mediated cisplatin uptake in PTECs, and thus could be indicative of the development of cisplatin-induced nephrotoxicity. We also demonstrated that cilastatin, a megalin antagonist, blocks the binding of cisplatin to megalin [12], and thereby could protect against cisplatin-induced nephrotoxicity [23]. Hence, it would be worthwhile to investigate whether blockade of cisplatin binding to megalin could prevent cisplatin-induced nephrotoxicity, particularly in patients with elevated urinary A-megalin levels.

The present study has several limitations. First, this was a single-institutional study with a limited number of patients. Further studies in different institutions and a large sample size are needed to verify our findings. Second, the relationship between urinary megalin levels and long-term renal dysfunction was not examined. Finally, we defined the adverse renal event as eGFR decline > 10 mL/min/1.73 m^2^ [20]. In clinical practice, the Kidney Disease Improving Global Outcomes criteria are often used as a definition of acute kidney injury; however, there were few patients who met the criteria in this study.

## Conclusions

This is the first report demonstrating that prechemotherapy urinary A-megalin levels are correlated with the development of cisplatin-induced nephrotoxicity and may be a novel predictor of nephrotoxicity. This has clinical implications for identifying patients at risk for cisplatin-induced nephrotoxicity and developing possible prophylactic therapies.

## Supplementary information

**Additional file 1: Table S1.** Chemotherapy regimens. **Table S2.** Baseline urinary markers. **Table S3.** Correlation matrix for all pairs of tested biomarkers.

## Data Availability

The datasets used and/or analyzed during the current study are available from the corresponding authors on reasonable request.

## Abbreviations

CI: Confidence interval
Cr: Creatinine
eGFR: Estimate glomerular filtration rate
ELISA: Enzyme-linked immunosorbent assay
HR: Hazard ratio
L-FABP: Liver-type fatty acid-binding protein
mAb: Monoclonal antibody
MEG: Megalin
MPM: Malignant pleural mesothelioma
NAG: *N*-acetyl-β-D-glucosaminidase
NGAL: Neutrophil gelatinase-associated lipocalin
NSAID: Non-steroidal anti-inflammatory drug
NSCLC: Non-small cell lung cancer
PTECs: Proximal tubule epithelial cells
RAS: Renin-angiotensin-aldosterone system
SCLC: Small cell lung cancer
SD: Standard deviation
α_1_-MG: α_1_-microglobulin
β_2_-MG: β_2_-microglobulin
