# Supplementary material for: Correlation of prechemotherapy urinary megalin ectodomain (A-megalin) levels with the development of cisplatin-induced nephrotoxicity: a prospective observational study
Source: BMC Cancer. 2019 Dec 2;19:1170. doi: 10.1186/s12885-019-6398-2 (PMC6889728; doi:10.1186/s12885-019-6398-2)
Supplement: Supplementary file 1 — Additional file 1: Table S1. Chemotherapy regimens. Table S2. Baseline urinary markers. Table S3. Correlation matrix for all pairs of tested biomarkers. [file 12885_2019_6398_MOESM1_ESM.docx]

Table S1. Chemotherapy regimens

|  | (mg/m^2^) |  | (mg/m^2^) |  | (mg/kg) | *n* |
| --- | --- | --- | --- | --- | --- | --- |
| Cisplatin | 75 | Pemetrexed | 500 |  |  | 11 |
|  | 75 | Pemetrexed | 500 | Bevacizumab | 15 | 7 |
|  | 80 | Etoposide | 100 |  |  | 12 |
|  | 80 | Gemcitabine | 1000 |  |  | 3 |
|  | 75 | Gemcitabine | 1250 |  |  | 6 |
|  | 60 | Irinotecan | 60 |  |  | 3 |
|  | 80 | Docetaxel | 60 |  |  | 2 |
|  | 80 | Vinorelbine | 25 |  |  | 1 |

Table S2. Baseline urinary markers

| Urinary marker |  | Mean ± SD | Range |
| --- | --- | --- | --- |
| A-megalin | (pmol/g Cr) | 87.9 ± 46.6 | (1.6–203.6) |
| C-megalin | (pmol/g Cr) | 0.64 ± 0.76 | (0.0–3.2) |
| NAG | (IU/g Cr) | 9.20 ± 9.40 | (0.0–42.3) |
| α_1_-MG | (mg/g Cr) | 10.8 ± 13.0 | (0.8–60.0) |
| β_2_-MG | (μg/g Cr) | 299.6 ± 616.4 | (0.0–2762.8) |
| NGAL | (μg/g Cr) | 20.0 ± 37.4 | (0.0–198.0) |
| L-FABP | (μg/g Cr) | 3.4 ± 6.6 | (0.0–41.2) |

α_1_-MG, α_1_-microglobulin; β_2_-MG, β_2_-microglobulin; Cr, creatinine; L-FABP, Liver-type fatty acid-binding protein; NAG, N-acetyl-β-D-glucosaminidase; NGAL, neutrophil gelatinase-associated lipocalin; SD, standard deviation.

Table S3. Correlation matrix for all pairs of tested biomarkers

|  | eGFR | ΔeGFR | A-MEG | ΔA-MEG | C-MEG | ΔC-MEG | NAG | ΔNAG | α_1_-MG | Δα_1_-MG | β_2_-MG | Δβ_2_-MG | NGAL | L-FABP |
| --- | --- | --- | --- | --- | --- | --- | --- | --- | --- | --- | --- | --- | --- | --- |
| eGFR |  | −0.395 | 0.299 | 0.241 | 0.043 | 0.075 | −0.26 | −0.092 | −0.065 | −0.023 | −0.142 | −0.248 | −0.34 | 0.123 |
| ΔeGFR | ** |  | −0.458 | −0.242 | −0.208 | −0.179 | −0.142 | −0.235 | −0.287 | 0.231 | −0.048 | −0.038 | 0.167 | −0.074 |
| A-MEG | * | ** |  | 0.26 | 0.205 | 0.304 | 0.276 | 0.149 | 0.235 | 0.158 | 0.195 | 0.372 | −0.248 | 0.019 |
| ΔA-MEG |  |  |  |  | 0.473 | 0.717 | 0.501 | 0.319 | 0.232 | −0.059 | 0.381 | 0.218 | −0.61 | 0.11 |
| C-MEG |  |  |  | ** |  | 0.673 | 0.487 | 0.333 | 0.242 | −0.087 | 0.279 | 0.349 | 0.009 | 0.395 |
| ΔC-MEG |  |  | * | ** | ** |  | 0.529 | 0.418 | 0.226 | −0.101 | 0.349 | 0.474 | 0.08 | 0.025 |
| NAG |  |  |  | ** | ** | ** |  | 0.5 | 0.37 | −0.072 | 0.714 | 0.505 | 0.25 | 0.451 |
| ΔNAG |  |  |  | * | * | ** | ** |  | 0.069 | −0.173 | 0.243 | 0.425 | 0.053 | 0.14 |
| α_1_-MG |  |  |  |  |  | * |  |  |  | 0.094 | 0.44 | 0.188 | −0.114 | 0.093 |
| Δα_1_-MG |  |  |  |  |  |  |  |  |  |  | 0.042 | −0.074 | −0.258 | -0.018 |
| β_2_-MG |  |  |  | * |  | * | ** |  | ** |  |  | 0.465 | 0.059 | 0.167 |
| Δβ_2_-MG |  |  | * |  | ** | ** | ** | ** |  |  | ** |  | 0.043 | 0.046 |
| NGAL |  |  |  |  |  |  |  |  |  |  |  |  |  | 0.26 |
| L-FABP |  |  |  |  | ** |  | ** | ** |  |  |  |  |  |  |

α_1_-MG, α_1_-microglobulin; β_2_-MG, β_2_-microglobulin; eGFR, estimated glomerular filtration rate; L-FABP, Liver-type fatty acid-binding protein; MEG, megalin; NAG, N-acetyl-β-D-glucosaminidase; NGAL, neutrophil gelatinase-associated lipocalin. The upper area shows the correlation coefficient and the lower area shows a significant difference. **P* < 0.05; ***P* < 0.001. ΔeGFR = (minimum eGFR after cisplatin administration) – (eGFR before cisplatin administration). Δurinary marker = (maximum urinary marker after cisplatin administration) – (urinary marker before cisplatin administration). NGAL and L-FABP were measured only before cisplatin administration.
